# Derivation and validation of a cardiovascular risk score for prediction of major acute cardiovascular events in non‐alcoholic fatty liver disease; the importance of an elevated mean platelet volume

**DOI:** 10.1111/apt.15192

**Published:** 2019-03-05

**Authors:** Robin D. Abeles, Benjamin H. Mullish, Roberta Forlano, Torben Kimhofer, Maciej Adler, Alexandros Tzallas, Nikolaos Giannakeas, Michael Yee, Jamil Mayet, Robert D. Goldin, Mark R. Thursz, Pinelopi Manousou

**Affiliations:** ^1^ Liver Unit/ Division of Integrative Systems Medicine and Digestive Disease, Department of Surgery and Cancer St Mary’s Hospital Campus, Imperial College London London UK; ^2^ Department of Surgery and Cancer Centre for Computational and System Medicine, Imperial College London London UK; ^3^ Department of Computer Engineering, School of Applied Technology Technological Educational Institute of Epirus Arta Greece; ^4^ Department of Endocrinology St Mary’s Hospital, Imperial College Healthcare NHS Trust London UK; ^5^ National Heart and Lung Institute, Hammersmith Hospital, Imperial College London London UK; ^6^ Department of Cellular Pathology St Mary’s Hospital, Imperial College Healthcare NHS Trust London UK

## Abstract

**Background:**

Atherosclerotic cardiovascular disease is a key cause of morbidity in non‐alcoholic fatty liver disease (NAFLD) but appropriate means to predict major acute cardiovascular events (MACE) are lacking.

**Aim:**

To design a bespoke cardiovascular risk score in NAFLD.

**Methods:**

A retrospective derivation (2008‐2016, 356 patients) and a prospective validation (2016‐ 2017, 111 patients) NAFLD cohort study was performed. Clinical and biochemical data were recorded at enrolment and mean platelet volume (MPV), Qrisk2 and Framingham scores were recorded one year prior to MACE (Cardiovascular death, acute coronary syndrome, stroke and transient ischaemic attack).

**Results:**

The derivation and validation cohorts were well‐matched, with MACE prevalence 12.6% and 12%, respectively. On univariate analysis, age, diabetes, advanced fibrosis, collagen proportionate area >5%, MPV and liver stiffness were associated with MACE. After multivariate analysis, age, diabetes and MPV remained independently predictive of MACE. The “NAFLD CV‐risk score” was generated using binary logistic regression:

0.06*(Age) + 0.963*(MPV) + 0.26*(DM^1^) – 16.44;

^1^Diabetes mellitus: 1: present; 2: absent.

(AUROC 0.84). A cut‐off of −3.98 gave a sensitivity 97%, specificity 27%, PPV 16%, and NPV 99%. An MPV alone of >10.05 gave a sensitivity 97%, specificity 59%, PPV 24% and NPV 97% (AUROC 0.83). Validation cohort AUROCs were comparable at 0.77 (NAFLD CV‐risk) and 0.72 (MPV). In the full cohort, the NAFLD CV‐risk score and MPV outperformed both Qrisk2 and Framingham scores.

**Conclusions:**

The NAFLD CV risk score and MPV accurately predict 1‐year risk of MACE, thereby allowing better identification of patients that require optimisation of their cardiovascular risk profile.

## 1 INTRODUCTION

Non‐alcoholic fatty liver disease (NAFLD) is estimated to affect 25% of the world's population and represents a spectrum of liver disease that ranges from simple steatosis (SS) to steatohepatitis (NASH), found in 30%‐70% on biopsy, with or without fibrosis.^1^ Approximately 41% of patients with NASH will experience progression of liver fibrosis over time, with the associated risks of developing cirrhosis, liver failure and hepatocellular carcinoma.^2^ NASH is projected to become the leading indication for liver transplant in the USA by 2020.^2^ However, the leading causes for morbidity and mortality in patients with NAFLD are due to atherosclerotic cardiovascular complications, with patients who have NASH or advanced fibrosis being at greater risk than those with SS.^3^, ^4^

Various cardiovascular risk scoring systems are widely‐used in clinical practice including the Framingham^5^ and Qrisk2 Score.^6^ These estimate the 10‐year risk of atherosclerotic cardiovascular events (including acute coronary syndrome and stroke) and have been validated in large cohorts of the general population. However, patients with NAFLD may be considered of higher risk as NAFLD is associated with various markers of subclinical atherosclerosis^7^, ^8^ and high‐risk coronary disease.^9^ Furthermore, the Framingham risk score does not include key features of the metabolic syndrome (including obesity and insulin resistance), which are evidently important risk factors for atherosclerotic events in those with NAFLD.^10^ The standard cardiovascular screening calculations may therefore not perform as well in patients with NAFLD.

Platelet activation is a typical feature in the pathophysiology of a range of diseases, including inflammatory and vascular disorders.^11^ Larger platelets are metabolically and enzymatically more active than smaller platelets, with greater aggregability, and contain a greater amount of pro‐thrombotic material.^12^ As such, there is interest as to whether markers of platelet size and function may be a useful biomarker of activity of such disorders. Mean platelet volume (MPV) is provided with every complete blood count result and has been shown to be elevated in patients with atherothrombotic disease^13^ and insulin resistance.^14^

Although there are some conflicting data,^15^, ^16^ MPV has also been shown to be elevated in people with NAFLD.^17^, ^18^, ^19^, ^20^, ^21^, ^22^ Higher MPV levels are found in patients with more advanced fibrosis compared to earlier fibrosis, and in those with NASH compared to those without.^20^, ^22^

We aimed to investigate whether elevated MPV is associated with an increased risk of cardiovascular events in patients with NAFLD and whether its incorporation in a cardiovascular risk score for patients with NAFLD would identify patients at higher risk for major acute cardiovascular events (MACE) compared to current standard cardiovascular risk scores.

## 2 MATERIALS AND METHODS

### 2.1 Study population

We performed a retrospective derivation (from January 2008 to July 2016) and a prospective validation (from August 2016 to March 2017) for 1‐year prediction of MACE, enrolling all consecutive patients at their first appointment at the specialist NAFLD clinic, St. Mary's Hospital, Imperial College Healthcare NHS Trust. Inclusion criteria were a clinical (liver ultrasound scan consistent with fatty liver, controlled attenuation parameter [CAP] score >250 dB/m) or histological diagnosis of NAFLD. Exclusion criteria were the use of steatogenic drugs, excess alcohol consumption (defined as weekly consumption of more than 14 units of alcohol),^23^ as well as any other concomitant liver disease.

At the time of enrolment: demographic (gender, age, ethnicity, smoking habit), anthropometric (body mass index, waist circumference) and biochemical data (liver function tests, full blood count, fasting lipids, HbA_1_c, ferritin, coagulation) were recorded. Ethnicities were clustered into 6 groups: Caucasian, Arab, Hispanic and Latino, South Asian, East Asian and African/Afro‐Caribbean. Smoking‐level was categorised as (a) nonsmokers, (b) ex‐smokers, (c) light smokers (<10 cigarettes/day), (d) moderate smokers (10‐20 cigarettes/day) and (e) heavy smokers (>20 cigarettes/day).^24^ Hypertension was recorded as present if documented in their medical records; it was noted that some patients were taking anti‐hypertensive medication for nonhypertensive indications. Diabetes mellitus was recorded as present if documented in the patients’ medical records.

Cardiovascular death, acute coronary syndrome (ACS), stroke, and transient ischaemic attack (TIA) were defined as major acute cardiovascular events (MACE). ACS was defined as a diagnosis of STEMI, type 1 NSTEMI and/or unstable angina. MACE were adjudicated by two researchers independently reviewing the medical records of included patients. Cardiovascular death was defined as death resulting from ACS or stroke as primary cause. MPV was recorded either 1‐year prior to a MACE or at baseline. For each patient, cardiovascular risk was estimated using Qrisk2 score^25^ and the Framingham score, using the sex‐specific equations of Wilson.^26^ All included patients were monitored in our specialist NAFLD clinic at least once every 6 months for more than 12 months, to ensure a comprehensive collection of clinical data.

### 2.2 Histology

Liver biopsies were performed for standard clinical indications. Liver biopsy specimens were formalin‐fixed, paraffin‐embedded, stained with Hematoxylin & Eosin (H&E) and Sirius Red and were scored by an experienced liver pathologist (RG) as per the NASH CRN scoring system.^27^ Biopsies were deemed to have definite NASH if the NAS score was ≥5, probable NASH if NAS 3‐4, and no NASH if <3.

Quantitation of fat percentage and Collagen Proportionate Area (CPA), was performed using an automated image analysis recently validated by our group.^28^ A value of CPA > 5% was considered as advanced fibrosis (F3).

### 2.3 Ethics

This research has been supported by the NIHR Imperial BRC. The Imperial Hepatology and Gastroenterology Biobank is fully REC approved by Oxford C Research Ethics Committee under REC reference 16/SC/0021.

### 2.4 Statistical analysis

The distribution of variables was explored using the Shapiro‐Wilk test and were normally distributed. Descriptive statistics were computed for all variables, with continuous variables expressed as means and standard deviation (SD), and categorical variables expressed as relative frequencies and percentages. Univariate analysis (by Student's *t* test and ANOVA for continuous, and chi‐square test for categorical variables respectively), with Bonferroni correction, was used to identify the variables significantly associated with a 1‐year risk of MACE. Significant variables were carried forward to univariate and multivariate Cox regression analysis to identify the hazard ratios (HR) of the variables independently associated with a 1‐year risk of MACE.

Binary logistic regression was then used to generate a formula for the prediction of 1‐year risk of MACE. The Brier Score was used to assess the accuracy of the prediction of the derived formula with values ranging from 0 (best accuracy) to 1 (lowest accuracy). Furthermore, the Hosmer‐Lemeshow test was calculated to estimate the goodness of fit for the logistic regression model with values ranging from 0 (lowest fit) to 1 (best fit).

ROC (receiver operating characteristic) curves were used to assess the diagnostic performance of this new algorithm and MPV compared to the established cardiovascular risk scoring systems. Areas under ROC curve (AUROC) with 95% confidence intervals were calculated under nonparametric (distribution free) assumption. Optimal cut‐off values were calculated to maximise sensitivity and specificity. For each cut‐off, sensitivity, specificity, positive predictive value (PPV) and negative predictive value (NPV) were reported based upon the observed prevalence of MACE within the population. Finally, pairwise statistical comparison of AUROCs was performed using the DeLong method between NAFLD CV risk score and traditional CV risk scores.

All tests were two‐sided and a *P* value 0.05 was considered significant. Statistical analysis was performed using SPSS^©^ (version 24.0; SPSS Inc Chicago, IL).

## 3 RESULTS

### 3.1 Derivation group

Three hundred and sixty five patients, with a median follow‐up of 36 months (18‐76) were included in the derivation group: 232 (65%) were male and 139 (39%) had hypertension. Diabetes mellitus was diagnosed in 183 (51%) patients, of whom 14% were diet controlled, 70% were on oral antidiabetic drugs and/or injectable GLP1 analogues, and 16% were on insulin treatment. Individual smoking‐level data was available for 305 (83%) patients, showing that 24 (7%) patients were active smokers (smoking level 3‐5) and 26 (7%) ex‐smokers. Mean MPV was 10.6 ± 1.4 fL, mean BMI 30.6 ± 4.6 kg/m^2^, mean liver stiffness 9.4 ± 8.4 kPa and mean CAP score 317 ± 55 dB/m. 41 (11%) patients were at moderate or high CV risk according to the Framingham score, and the mean Qrisk2 score was 12.9% ± 11.8 (Table S1).

Forty‐five (12.6%) patients experienced a MACE (39 ACS, 2 Stroke, 4 TIA) from which 3 died. Patients who experienced an MACE had higher MPV (12.2 vs 10.4 fL, *P* < 0.001) and liver stiffness (9.4 vs 6.4 kPa, *P* = 0.049) values compared to those who did not experience a MACE. The proportion of patients with diabetes mellitus (66% vs 48%, *P* = 0.028), hypertension (66% vs 35%, *P* < 0.001), on anti‐hypertensive treatment (73% vs 39%, *P* = 0.013) and on aspirin (42% vs 7%, *P* = 0.045) was higher in patients who experienced a MACE compared to those who did not. The Qrisk2 score was higher in patients who experienced a MACE (22.5 vs 11.5, *P* < 0.001) but not the Framingham score (Table 1).

**Table 1:** Clinical characteristics of the derivation and validation group: differences between the subgroups with and without acute cardiovascular event (MACE)

Variable	Derivation group, n = 365		
	With MACE, n = 45	No MACE, n = 320	*P*‐value
Age (y)	58 ± 9.3	49.6 ± 12.8	0.045
Male gender (%)	27 (60)	205 (64)	0.43
Diabetes mellitus (%)	30 (66)	153 (48)	0.028
Hypertension (%)	25 (66)	114 (35)	0.001
Ethnic groups			
Caucasian (%)	25 (56)	149 (46)	0.18
Arab (%)	8 (18)	39 (12)	0.33
Hispanic and Latino (%)	5 (11)	27 (9)	0.59
South Asian (%)	5 (11)	67 (12)	0.21
East Asian (%)	1 (2)	28 (8)	0.13
African/Afro‐Caribbean (%)	1 (2)	10 (3)	0.72
Smoking‐level			
Nonsmokers (%)	36 (80)	219 (69)	0.33
Ex‐smokers (%)	4 (9)	22 (49)	0.81
Light smokers (%)	1 (2)	6 (13)	0.32
Moderate smokers (%)	1 (2)	3 (1)	0.48
Heavy smokers (%)	3 (7)	10 (3)	0.001
BMI (kg/m^2^)	31.3 ± 5.1	30.5 ± 4.5	0.64
BMI>25.1 kg/m^2^ (%)	42 (93)	292 (91)	0.88
BMI>30.1 kg/m^2^(%)	21 (47)	139 (43)	0.8
BMI>35.1 kg/m^2^(%)	8 (18)	50 (16)	0.51
BMI>40.1 kg/m^2^(%)	3 (7)	14 (5)	0.52
Total cholesterol (mmol/L)	4.4 ± 1	4.5 ± 1.1	0.99
HDL (mmol/L)	1 ± 0.25	1.3 ± 4.2	0.98
LDL (mmol/L)	2.4 ± 0.9	2.5 ± 1	0.96
Triglycerides (mmol/L)	2 ± 1	2 ± 1.2	0.64
HbA1c (mmol/L)	47 ± 13.9	49.5 ± 27.8	0.77
AST (IU/L)	49.9 ± 34	49.9 ± 34	0.42
ALT (IU/L)	59.7 ± 35.4	74.2 ± 47.4	0.78
Albumin (g/L)	39.9 ± 3.4	40.9 ± 3.4	0.93
Platelet (10^9^/L)	219 ± 68	228.5 ± 70	0.85
MPV (fL)	12.2 ± 1.1	10.4 ± 1.3	0.001
Q‐risk 2 score (%)	22.5 ± 13.1	11.5 ± 10.9	0.001
Framingham score	11 ± 3	7 ± 5	0.45
NAFLD CV risk score	‐0.8 ± 1.4	‐2.9 ± 1.5	0.003
Stiffness (kPa)	9.4 ± 8.4	6.4 ± 8.4	0.049
Stiffness >7.1 kPa (%)	22 (48)	113 (31)	0.048
CAP score (dB/m)	322 ± 60	311 ± 56	0.32
CAP score >250 dB/m (%)	24 (53)	193 (60)	0.53
Use of antihypertensive (%)	33 (73)	126 (39)	0.013
Use of statin (%)	29 (64)	151 (47)	0.06
Use of aspirin (%)	19 (42)	23 (7)	0.045

ALT, alanine aminotransferase; AST, aspartate aminotransferase; BMI, body mass index; CAP, controlled attenuation parameter; HDL, high density lipoprotein; LDL, low density lipoprotein; MACE, major acute cardiovascular event; MPV, mean platelet volume.

231 (65%) patients in the derivation group underwent a liver biopsy, 24 (10%) of whom experienced a MACE. The presence of advanced fibrosis (stage 3‐4) and CPA > 5% were higher in patients who experienced MACE compared to those that did not (71% vs 43%, *P* = 0.023 and 41% vs 28%, *P* = 0.047). Fat % and steatosis grade, lobular inflammation and ballooning score, as well as the presence of definite or probable NASH, were not significantly different between the subgroups (Table 2).

**Table 2:** Histological characteristics of patients undergoing liver biopsy in derivation group: differences between those that experienced and MACE and those that did not

Variable	Derivation group, n = 231		
	With MACE, n = 24	No MACE, n = 207	*P*‐value
Fibrosis stage			
F0 (%)	1 (4)	22 (10)	0.06
F1‐2 (%)	6 (25)	97 (47)	0.61
F3‐4 (%)	17 (71)	88 (43)	0.023
Steatosis grade			
Mild (%)	7 (30)	60 (29)	0.66
Moderate (%)	11 (46)	118 (57)	0.051
Severe (%)	6 (25)	29 (14)	0.44
Lobular Inflammation			
None	8 (33)	72 (35)	0.44
<2 foci (%)	10 (42)	101 (48)	0.21
2‐4 foci (%)	5 (21)	32 (16)	0.72
>4 foci (%)	1 (4)	2 (1)	0.08
Ballooning score			
None (%)	6 (25)	50 (23)	0.52
Few ballooned cells (%)	11 (46)	101 (49)	0.88
Many ballooned cells (%)	7 (29)	56 (28)	0.79
Definite or probable NASH (%)	18 (75)	166 (80)	0.53
Non‐NASH (%)	6 (25)	41 (20)	0.63
Fat%	7.2 ± 3	10.5 ± 6	0.55
Fat % >5 (%)	13 (54)	142 (61)	0.28
Fat % >10 (%)	8 (33)	102 (48)	0.051
Fat % >15 (%)	2 (8)	40 (18)	0.06
CPA, %	7 ± 5.3	4.9 ± 4.7	0.39
CPA >2 (%)	14 (58)	134 (64)	0.67
CPA >5 (%)	10 (41)	60 (28)	0.047
CPA >12 (%)	2 (8)	16 (7)	0.65

CPA, collagen proportionate area; MACE, major acute cardiovascular event; NASH, non‐alcoholic steatohepatitis.

### 3.2 NAFLD Cardiovascular‐risk score

On univariate analysis, age, presence of diabetes mellitus, advanced fibrosis (F3‐F4), CPA > 5%, liver stiffness and MPV were associated with a 1‐year risk of MACE. However, after multivariate analysis, only age (HR 1.12, 1.01‐1.23, *P* = 0.01), presence of diabetes (HR 1.9, 1.1‐2.7, *P* = 0.002) and MPV (HR 2.9, 1‐3.7, *P* = 0.02) remained independently associated with 1‐year risk of MACE (Table 3).

**Table 3:** Predictive factors for acute cardiovascular event in people with Non‐alcoholic fatty liver disease by univariate and multivariate Cox‐regression analysis

Factor	Univariate analysis, HR (95% CI), *P* value	Multivariate analysis, HR (95% CI), *P* value
Age	1.06 (1.03‐1.09), *P* = 0.001	1.12 (1.01‐1.23), *P* = 0.01
Diabetes mellitus	2.06 (1.07‐3.99), *P* = 0.038	1.9 (1.1‐2.7), *P* = 0.002
Hypertension	0.51 (0.28‐1.2), *P* = 0.32	NS
Heavy smoking	1.12 (0.93‐1.39), *P* = 0.071	NS
MPV	3.2 (2.3‐4.6), *P* = 0.001	2.9 (1.9‐3.7), *P* = 0.02
Stiffness	1.02 (1‐1.04), *P* = 0.037	NS
Liver Stiffness >7.1 kPa	1.01 (0.88‐1.34), *P* = 0.52	NS
F3‐F4	1.16 (1.08‐1.28), *P* = 0.038	NS
CPA >5%	1.1 (1.01‐1.23), *P* = 0.043	NS
Use of anti‐hypertensive	0.81 (0.56‐1.02), *P* = 0.053	NS
Use of aspirin	0.92 (0.67‐1.07), *P* = 0.072	NS

CI, confidence interval; CP, collagen proportionate area; MPV, mean platelet volume; NS, nonsignificant.

Using binary logistic regression, a formula was generated to predict acute CV events within one year. In this formula, termed the NAFLD CV‐risk score, the weight of each variable was assigned based on the β‐coefficient from the logistic regression analysis: 0.06*(Age) + 0.963 (*MPV) + 0.26*(DM^1^) – 16.44

^1^Diabetes mellitus: 1: present; 2: absent.

A free online tool to calculate the formula is available via the following link: https://ld-eye.com/index.php?r=site/CVDRiskScore.

In the derivation cohort, the NAFLD CV‐risk score ranged from −7.1 to 3.6. The mean NAFLD CV risk score was higher in the group with MACE compared to those without MACE, −0.8 ± 1.4 and −2.9 ± 1.5 respectively (*P* = 0.003). The overall Brier score was 0.08, indicating that the prediction of the formula was accurate. Moreover, the Hosmer‐Lemeshow test was 0.99, indicating that the derived model fits well.

**Figure 1:**
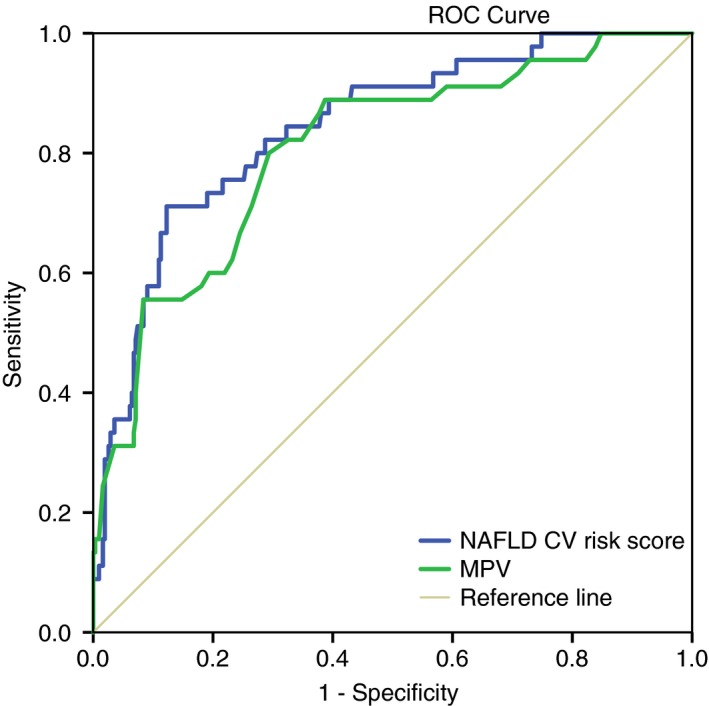
Areas under ROC curve (AUROCs) of NAFLD CV Risk score and MPV for predicting MACE in the derivation cohort. AUROCs were 0.84 (*P* = 0.001, 95% CI: 0.78‐0.91) for NAFLD CV Risk Score and 0.83 (*P* = 0.001, 95% CI: 0.77‐0.89) for MPV alone (AUROC, area under receiver operating characteristics, MPV, mean platelet volume, MACE, major acute cardiovascular events)

AUROCs for the prediction of MACE in the derivation cohort were 0.84 (*P* = 0.001, 95% CI = 0.78‐0.91) for NAFLD CV‐risk score and 0.83 (*P* = 0.001, 95% CI = 0.77‐0.89) for MPV alone (Figure 1). A cut‐off of NAFLD CV risk score of −3.98 gave a sensitivity 97%, specificity 27%, PPV 16% and NPV 99%. A cut‐off of MPV >10.05 gave a sensitivity 97%, specificity 59%, PPV 24% and NPV 97% (Table 4).

**Table 4:** Sensibility, specificity, NPV and PPV for cut‐offs predicting MACE in the derivation and validation group

	Sensitivity (%)	Specificity (%)	PPV (%)	NPV (%)
NAFLD CV risk score Cut‐off: −3.98				
Derivation group	97	27	16	99
Validation group	92	18	13	95
MPV Cut‐off: 10.05				
Derivation group	97	59	24	97
Validation group	84	24	20	94

MACE, major acute cardiovascular event; MPV, mean platelet volume; NAFLD CV risk Score, Non‐alcoholic fatty liver disease cardiovascular risk score; NPV, Negative predictive value; PPV, positive predictive value.

### 3.3 Validation of NAFLD CV score and MPV

One hundred and eleven patients were included in the validation cohort. 69 (62%) were male and 33 (29%) had hypertension. Diabetes mellitus was diagnosed in 54 (48%) patients, of whom 16% were diet controlled, 69% on oral antidiabetic drugs and/or non‐insulin injectables, and 15% on insulin treatment. Individual smoking‐level was available in 102 (91%) patients, showing that 6 (5%) patients were active smokers (smoking level 3, 4 or 4), while 14 (13%) ex‐smokers. Mean MPV was 11 ± 1.3 fL, mean BMI 30.5 ± 4.8 kg/m^2^, mean liver stiffness 8.9 ± 6.3 kPa and CAP score 319 ± 54 dB/m (Table S1). 15 (13%) patients were at moderate or high CV risk according to Framingham score, while mean Qrisk2 score was 12.5% ± 12.2.

Thirteen (12%) patients in the validation group experienced a MACE (10 ACS, 1 Stroke, 2 TIA) from which 1 died. 56 (50%) patients in the validation group underwent liver biopsy, of whom 7 (8%) experienced a MACE. There were no significant differences between the derivation and validation groups with regards to clinical variables. In the derivation group, a higher proportion of biopsies had mild fibrosis (F1‐2) (44% vs 26%, *P* = 0.004) but a lower proportion had mild steatosis (29% vs 44%, *P* = 0.03) than the validation group biopsies (Tables S1 and S2).

In the validation cohort, NAFLD CV risk score values ranged from −8.4 to 1.75. The mean NAFLD CV risk score was higher in the group with MACE compared to those without MACE, −1 ± 1.1 and −2.62 ± 1.4 respectively (*P* = 0.002). The overall Brier score was 0.2, indicating that the prediction of the formula was accurate. Moreover, the Hosmer‐Lemeshow test was 0.98, indicating that the derived model fits well.

**Figure 2:**
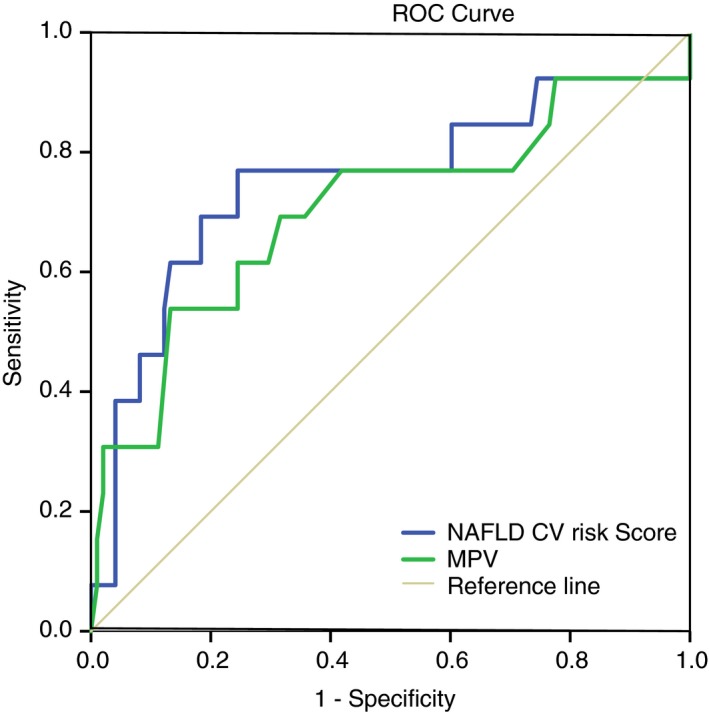
The AUROCs for the prediction of MACE in the validation cohort for NAFLD CV‐risk score and MPV alone were 0.77 (95% CI: 0.61‐0.94, *P* = 0.004) and 0.72 (0.52‐0.88, *P* = 0.018). (AUROC, area under receiver operating characteristics, MPV, mean platelet volume, MACE, major acute cardiovascular events)

The AUROCs for the prediction of MACE in the validation cohort for NAFLD CV‐risk score and MPV alone were 0.77 (95% CI: 0.61‐0.94, *P* = 0.004) and 0.72 (0.52‐0.88, *P* = 0.018) respectively (Figure 2). The cut‐off values derived from the derivation cohort for the NAFLD‐CV‐Risk score gave sensitivities of 92% and 84% for the NAFLD‐CV‐risk score and MPV alone, respectively, specificities of 18% and 24%, respectively, NPV of 95% and 94%, respectively and PPV of 13% and 20%, respectively (Table 4).

### 3.4 Comparison of NAFLD CV score and MPV vs traditional scores

**Figure 3:**
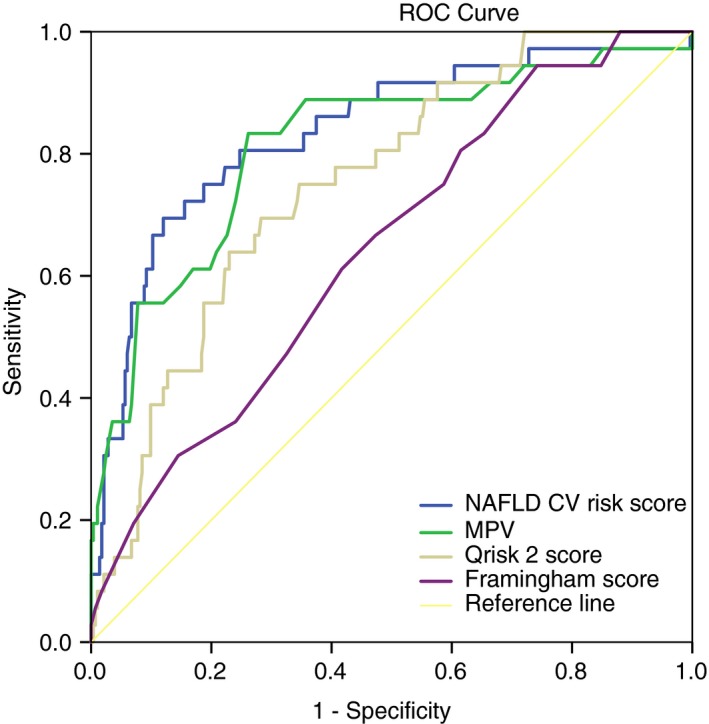
The AUROCs for the prediction of MACE were were 0.83 (*P* = 0.001, 95% CI: 0.77‐0.89) for NAFLD CV‐risk score, 0.78 (*P* = 0.001, 95% CI: 0.72‐0.85) for MPV, 0.73 (*P* = 0.005, 95% CI: 0.59‐0.89) for Qrisk2 score and 0.64 (*P* = 0.001, 95% CI: 0.55‐0.73) for Framingham score. (AUROC, area under receiver operating characteristics, MPV, mean platelet volume, MACE, major acute cardiovascular events)

The clinical, biochemical and histological differences between patients who experienced a MACE and those that did not for the whole study cohort are presented in Tables S3 and S4. In the whole study population, the NAFLD CV‐risk score ranged from −8.4 to 3.6. The mean NAFLD CV risk score was higher in the group with MACE compared to those without MACE, −0.97 ± 1.6 and −2.8 ± 1.5, respectively (*P* = 0.002). AUROCs for the prediction of MACE were 0.83 (*P* = 0.001, 95% CI=0.77‐0.89) for NAFLD CV‐risk score, 0.78 (*P* = 0.001, 95% CI=0.72‐0.85) for MPV, 0.73 (*P* = 0.005, 95% CI=0.59‐0.89) for Qrisk2 score and 0.64 (*P* = 0.001, 95% CI=0.55‐0.73) for Framingham score (Figure 3). The DeLong method revealed that the AUROC of NAFLD CV risk score was significantly higher when compared to MPV (*P* = 0.005), Qrisk2 Score (*P* = 0.042) and Framingham score (*P* = 0.003).

## 4 DISCUSSION

We have demonstrated that the prevalence of MACE in our cohort of NAFLD patients is 12%, which is double the age standardised prevalence of MACE in the UK.^29^ We have presented a prospectively validated cardiovascular risk score algorithm in NAFLD with a result of more than −3.98 predicting a 16%‐27% 1‐year chance of suffering a MACE and a result lower than −3.98 giving a 97%‐99% accurate negative predictive value. Finally, we have highlighted the clinical significance of a raised MPV in this patient cohort.

Our study has several strengths. Firstly, our patient cohort was well selected for NAFLD, as all patients had negative non‐invasive liver screens, underwent transient elastography, and 60% of them had confirmatory liver biopsies. The derivation and validation cohorts were also broadly well‐matched in terms of epidemiological, clinical and histological parameters. The cohort consisted of a real‐world population with the full range of features of metabolic syndrome and contained a wide mix of ethnicities that are typically representative of large urban centres. Secondly, we chose ‘hard’ end points for classifying MACE (ACS, stroke and TIA) to ensure that MACE rates were not over‐estimated. A limitation of our study is that all patients were recruited from a tertiary level specialist NAFLD clinic and so this may lead to a selection bias towards a more high‐risk population. Indeed, 50% of our biopsied patients had advanced fibrosis, much higher than would be seen in general secondary or primary care clinics. Nonetheless, our data re‐emphasise the importance of assessing cardiovascular risk for patients with NAFLD within a specialist clinic and further validation within a secondary or primary care setting is warranted. A further limitation is that we only quantified alcohol intake of included patients using clinical assessment (rather than a validated questionnaire of alcohol intake), and it is therefore possible that a small proportion of included patients had alcohol use as a contributory factor to their liver disease.

In the general population, the risk of atherosclerotic cardiovascular disease is estimated using one of the established scoring systems, such as the Qrisk2 or Framingham, to guide primary prevention with pharmacological therapy,^30^ whilst interventional therapy is not undertaken routinely for primary prophylaxis. A previous study demonstrated that the Framingham score had utility in predicting coronary heart disease in NAFLD patients,^31^ but assessment for other MACE was not performed. MELD‐Na has also been shown to have a potential role in predicting MACE in NAFLD patients, but FIB‐4 (rather than histology) was used to assess fibrosis stage in this study, limiting interpretability.^32^ There has also been the recent demonstration that the atherogenic index of plasma (AIP; an established risk factor for cardiovascular disease) in those with abnormal metabolic profiles compared to those with normal profiles, which is likely to also be of relevance to NAFLD.^33^ Our novel algorithm outperforms the established scoring systems, however, it is accepted that the established scores were developed to identify a 10% risk of MACE at 10 years rather than 1‐year risk.^31^ Nevertheless, given the high prevalence of MACE in NAFLD, our data would certainly support the importance of assessing 1‐year risk using the NAFLD CV Risk Score and ensuring that high risk patients are on primary prophylaxis.

Interestingly, a significant proportion of our patients who suffered from a MACE were not taking statins, anti‐hypertensive medications or aspirin. It should also be noted that a higher proportion of patients who were on these medications still experienced a MACE compared to those that were not on these medications. Therefore, it could be argued that these patients would benefit from enhanced cardiovascular risk assessment and referral to a cardiologist.

Our data are consistent with those previously published with regards to the major risk factors associated with the development of MACE, namely age, heavy smoking, and the presence of hypertension or diabetes.^34^ In contrast with published data that suggests the degree of steatosis, as defined by ultrasound criteria, is associated with an increased risk of cardiovascular events,^35^, ^36^, ^37^ we did not find any association between the severity of histological steatosis and cardiovascular risk. Consistent with Ekstedt et al and others^4^, ^38^ we found that only advanced fibrosis (defined histologically or by transient elastography) was associated with an increased cardiovascular risk but not the severity of NASH (as defined by NAS score or its constituent components). In addition, consistent with previous data,^39^ transaminases were not associated with an elevated risk of MACE.

A significant novel finding is the association between elevated MPV and cardiovascular risk in NAFLD, with a hazard ratio of 2.9 (1.9‐3.7) after multivariate analysis. MPV alone had an AUROC of 0.83 and a cut‐off of 10.05 with good positive and negative predictive values (20%‐24% and 94%‐97%, respectively) that were further enhanced with the additional clinical parameters in the NAFLD CV‐Risk score. Given the widespread availability of MPV as part of the standard full blood count, this single variable could be used as a simple and cheap initial screening tool by primary and secondary care physicians.

In the general population, raised MPV levels (but not platelet number) are associated with coronary artery disease events, including acute MI, as well increased rates of restenosis after MI.^40^, ^41^ The risk of stroke also appears to increase as MPV increases, as does an increased likelihood of larger volumes of cerebral damage,^42^, ^43^ together with the risk of early death in the early post‐stroke period.^40^ Raised MPV also has a strong and independent association with venous thromboembolic disease, even in the absence of trauma, surgery, immobilisation or malignancy.^44^ Furthermore, elevated MPV has also been showed to be associated with higher overall mortality within a population of patients requiring haemodialysis, a group who are at particularly high risk of atherosclerotic cardiovascular events.^45^

A clear future direction of interest for this work will be to establish greater mechanistic understanding of the association between the level of MPV and NAFLD's stage and cardiovascular complications, which may shed fresh insight into the pathophysiology of the condition. There have been several proposed mechanisms to explain the link between raised MPV level and cardiovascular events, in particular that larger platelets contain a higher density of prothrombotic material, encouraging the release of substances that amplify platelet activation, platelet adhesion and vascular neointimal proliferation, such as thromboxane A2.^46^ Larger platelets also demonstrate greater aggregability^47^, ^48^; furthermore, they also express a greater density of glycoprotein Ib and IIb/IIIa adhesion receptors, and display more reticulation, both being factors associated with a worse response to anti‐platelet therapy.^49^, ^50^

There are likely to be several factors contributing to the relationship between elevated MPV levels and the presence of advanced fibrosis in NAFLD. Firstly, insulin resistance may have a direct effect on platelet function per se: MPV levels are higher in non‐obese, normoglycaemic people with insulin resistance than matched people without,^14^ with insulin resistance causing reduced platelet sensitivity to the anti‐aggregating effects of insulin.^51^, ^52^ In addition, there appears to be a relationship between inflammation, platelet activity and hepatic fibrosis. Specifically, cytokines are key mediators of hepatic inflammation, and cytokines derived from the adipose tissue appear to play a key role in the progression of NAFLD.^53^ NAFLD is associated with an increase in inflammatory cytokines (including IL‐1, IL‐6 and TNF‐α), with cytokine plasma levels in NAFLD related to hepatic fat content, the degree of inflammation, and the extent of hepatic fibrosis.^53^ It has been previously suggested that adipose and a dysfunctioning epithelium may affect the bone marrow to produce larger platelets via cytokine‐driven mechanisms, with the characteristic cytokine profile found in people with NAFLD therefore affecting platelet size in people with the condition.^54^ Given the apparent association between platelet activity, inflammation and hepatic fibrosis, a limitation of our study is that inflammatory markers (including C‐reactive protein and ESR) were not available for all patients, so could not be considered in the development of our algorithm. Another future area of interest would be the exploration as to whether integration of such inflammatory markers into the NAFLD CV risk score may be of additional utility for the prediction of MACE in patients with NAFLD.

In summary, patients with NAFLD and a NAFLD CV score of more than −3.98 or a MPV greater than 10.05 are at a high risk of experiencing a MACE within 12 months. 1‐year cardiovascular risk is related to fibrosis stage rather than steatohepatitis. Physicians should, therefore, ensure that these patients are on appropriate primary prevention strategies and strongly consider referral for formal cardiovascular assessment. Understanding the pathophysiological mechanisms that underlie the association between an elevated MPV and cardiovascular risk may provide a novel target for drug development.

## AUTHORSHIP

*Guarantor of the article*: Dr Pinelopi Manousou is guarantor of this article.

*Author contributions*: RDA, BHM, MRT and PM originated the idea for the manuscript. RDG performed histological analysis. NG and AT performed the automated quantitation. RF and TK performed statistical analysis. RDA, BHM, RF, TK, MA and PM analysed the data and drafted the text. All authors read and approved the final draft submitted.
