# Supplementary material for: Derivation and validation of a cardiovascular risk score for prediction of major acute cardiovascular events in non‐alcoholic fatty liver disease; the importance of an elevated mean platelet volume
Source: Aliment Pharmacol Ther. 2019 Mar 5;49(8):1077–85. doi: 10.1111/apt.15192 (PMC6519040; doi:10.1111/apt.15192)
Supplement: Supplementary file 1 [file APT-49-1077-s001.docx]

**Supplemental Table 1. Clinical characteristics of the derivation and validation group: differences between the groups.**

| **Variable** | **Derivation group**  **n= 365** | **Validation group**  **n= 111** | **p- value** |
| --- | --- | --- | --- |
| **Age (years)** | 50.6 ± 12.7 | 51.1 ±13.6 | 0.46 |
| **Male gender (%)** | 232 (65) | 69 (62) | 0.63 |
| **Diabetes Mellitus (%)** | 183 (51) | 54 (48) | 0.72 |
| **Hypertension (%)** | 139 (39) | 33 (29) | 0.12 |
| **BMI (kg/m^2^)** | 30.6 ± 4.6 | 30.5 ± 4.8 | 0.68 |
| **T-Cholesterol (mmol/l)** | 4.5 ± 1.1 | 5.1 ± 2.8 | 0.63 |
| **HDL (mmol/l)** | 1.3 ± 3.9 | 1.2 ± 0.5 | 0.95 |
| **LDL (mmol/l)** | 2.5 ± 1 | 2.9 ± 1 | 0.05 |
| **Triglycerides (mmol/l)** | 2 ± 1.2 | 2.1 ± 1.6 | 0.28 |
| **HbA1c (mmol/l)** | 49.2 ± 26.5 | 47.1 ± 21.4 | 0.68 |
| **AST (IU/L)** | 49.9 ± 34 | 51.4 ± 26.3 | 0.91 |
| **ALT (IU/L)** | 72.4 ± 46.3 | 85.9 ± 59.9 | 0.06 |
| **Albumin (g/l)** | 40.7 ± 3.4 | 40.6 ± 3.1 | 0.51 |
| **Platelet (10^9^ /l)** | 227.3 ± 70.3 | 240 ± 79 | 0.18 |
| **Ferritin (µg/l)** | 287.4 ± 477 | 228.4 ± 231.2 | 0.84 |
| **MPV (fL)** | 10.6 ± 1.4 | 11 ± 1.3 | 0.06 |
| **Q-risk 2 score** | 12.9 ± 11.8 | 12.5 ± 12.2 | 0.73 |
| **Framingham score** | 8.1 ± 5 | 9.1 ± 5 | 0.17 |
| **Stiffness (kPa)** | 9.4 ± 8.4 | 8.9 ± 6.3 | 0.75 |
| **CAP score (dB/m)** | 312 ± 57 | 310 ± 63 | 0.65 |
| **MACE (%)** | 45 (12.6) | 13 (12) | 0.79 |
| **Use of Antihypertensive (%)** | 139 (38) | 36 (32) | 0.09 |
| **Use of statin (%)** | 180 (49) | 37 (37) | 0.32 |
| **Use of aspirin (%)** | 42 (11) | 13 (11) | 0.91 |

MACE Major Acute Cardiovascular Event, BMI Body Mass Index, HDL High Density Lipoprotein, LDL Low Density Lipoprotein, AST Aspartate Aminotransferase, ALT Alanine Aminotransferase, MPV Mean Platelet Volume, CAP Controlled Attenuation Parameter

**Supplemental Table 2. Histological characteristics of the derivation and validation group: differences between the groups.**

| **Variable** | **Derivation group**  **n= 231** | **Validation group**  **n= 56** | **p- value** |
| --- | --- | --- | --- |
| **Fibrosis stage** | | | |
| **F 0 (%)** | 23 (10) | 8 (14) | 0.44 |
| **F 1-F2 (%)** | 103 (44) | 14 (26) | **0.004** |
| **F 3-4 (%)** | 105 (46) | 32 (57) | 0.08 |
| **Steatosis Grade** | | | |
| **Mild (%)** | 67 (29) | 25 (44) | **0.03** |
| **Moderate (%)** | 129 (55) | 27 (48) | 0.89 |
| **Severe (%)** | 35 (15) | 4 (7) | 0.09 |
| **Lobular Inflammation** |  |  |  |
| **None** | 80 (35) | 16 (29) | 0.34 |
| **<2 foci (%)** | 111 (48) | 30 (53) | 0.57 |
| **2-4 foci (%)** | 37 (16) | 10 (18) | 0.62 |
| **>4 foci (%)** | 3 (1) | 0 (0) | 0.71 |
| **Ballooning score** |  |  |  |
| **None (%)** | 56 (25) | 12 (21) | 0.8 |
| **Few ballooned cells (%)** | 112 (48) | 30 (54) | 0.23 |
| **Many ballooned cells (%)** | 63 (27) | 14 (25) | 0.38 |
| **Definite or probable NASH (%)** | 184 (79) | 41 (73) | 0.45 |
| **Non-NASH (%)** | 47 (21) | 15 (27) | 0.61 |
| **Fat%** | 10.2 ± 6.2 | 9 ± 7.2 | 0.14 |
| **CPA%** | 5.1 ± 4.8 | 6.8 ±8.2 | 0.28 |

MACE Major Acute Cardiovascular Event, NASH Non-alcoholic Steatohepatitis, CPA Collagen Proportionate Area

**Suppl. table 3. Clinical characteristics of the whole study population: differences between subgroups with and without MACE.**

| **Variable** | **With MACE**  **n= 58** | **No MACE**  **n= 409** | **p- value** |
| --- | --- | --- | --- |
| **Age (years)** | 58.2 ± 9.8 | 49.8 ± 12.9 | **0.045** |
| **Male gender (%)** | 40 (68) | 292 (71) | 0.85 |
| **Diabetes Mellitus (%)** | 38 (54) | 199 (48) | **0.003** |
| **Hypertension (%)** | 32 (55) | 140 (34) | **0.001** |
| **BMI (kg/m^2^)** | 31.4 ± 5 | 30.4 ± 4.6 | 0.86 |
| **T-Cholesterol (mmol/l)** | 4.3 ± 1.1 | 5.7 ± 9.2 | 0.99 |
| **HDL (mmol/l)** | 1 ± 0.3 | 1.8 ± 6.1 | 0.9 |
| **LDL (mmol/l)** | 2.3 ± 0.9 | 3.2 ± 6.5 | 0.63 |
| **Triglycerides (mmol/l)** | 2.1 ± 1.8 | 2.7 ± 7.2 | 0.67 |
| **HbA1c (mmol/l)** | 48 ± 14.1 | 48.8 ± 26.1 | 0.71 |
| **AST (IU/L)** | 40 ± 22.1 | 52.8 ± 47.5 | 0.53 |
| **ALT (IU/L)** | 64.3 ± 47.8 | 82.1 ± 76 | 0.89 |
| **Albumin (g/l)** | 42.7 ± 2.7 | 39.6 ± 1.1 | 0.07 |
| **Platelet (10^9^ /l)** | 237.2 ± 58.3 | 245 ± 68 | 0.53 |
| **Ferritin (µg/l)** | 99.2 ± 142 | 279 ± 249 | 0.37 |
| **MPV (fL)** | 11.9 ± 1.3 | 10.6 ± 1.4 | **0.001** |
| **Q-risk 2 score** | 23.9 ± 12.9 | 11.3 ± 11 | **0.001** |
| **Framingham score** | 10.8 ± 4 | 8.2 ± 8 | **0.03** |
| **NAFLD CV risk score** | -0.97 ± 1.6 | -2.8 ± 1.5 | **0.002** |
| **Stiffness (kPa)** | 12 ± 13.6 | 8.9 ± 7 | **0.035** |
| **CAP score (dB/m)** | 325 ± 59 | 310 ± 58 | 0.23 |
| **Use of anti-hypertensive (%)** | 40 (69) | 155 (38) | **0.03** |
| **Use of statin (%)** | 33 (57) | 184 (45) | **0.05** |
| **Use of aspirin (%)** | 26 (45) | 29 (7) | **0.03** |

MACE Major Acute Cardiovascular Event, BMI Body Mass Index, HDL High Density Lipoprotein, LDL Low Density Lipoprotein, AST Aspartate Aminotransferase, ALT Alanine Aminotransferase, MPV Mean Platelet Volume, CAP Controlled Attenuation Parameter

**Suppl. Table 4. Histological characteristics of the subgroup undergoing liver biopsy in the study population: differences between subgroups with and without MACE.**

| **Variable** | **With MACE**  **n= 31** | **No MACE**  **n= 256** | **p- value** |
| --- | --- | --- | --- |
| **Fibrosis stage** |  |  |  |
| **F 0 (%)** | 2 (6) | 31 (12) | 0.52 |
| **F 1-2 (%)** | 8 (25) | 109 (42) | 0.13 |
| **F 3-4 (%)** | 21 (68) | 116 (45) | **0.047** |
| **Steatosis Grade** |  |  |  |
| **Mild (%)** | 10 (32) | 82 (32) | 0.73 |
| **Moderate (%)** | 15 (49) | 139 (54) | 0.43 |
| **Severe (%)** | 6 (19) | 35 (13) | 0.06 |
| **Lobular Inflammation** |  |  |  |
| **None** | 12 (38) | 89 (35) | 0.59 |
| **<2 foci (%)** | 12 (39) | 120 (46) | 0.42 |
| **2-4 foci (%)** | 6 (19) | 41 (16) | 0.78 |
| **>4 foci (%)** | 1 (3) | 6 (2) | 0.8 |
| **Ballooning score** |  |  |  |
| **None (%)** | 6 (19) | 64 (25) | 0.63 |
| **Few ballooned cells (%)** | 17 (55) | 121 (47) | 0.59 |
| **Many ballooned cells (%)** | 8 (26) | 71 (27) | 0.88 |
| **Definite or probable NASH (%)** | 22 (71) | 203 (79) | 0.68 |
| **Non-NASH (%)** | 9 (29) | 53 (21) | 0.07 |
| **Fat%** | 6.9 ± 3.6 | 10.4 ± 6.5 | 0.83 |
| **CPA%** | 6.7 ± 5.2 | 5.2 ± 5.6 | 0.078 |

MACE Major Acute Cardiovascular Event, NASH Non-alcoholic Steatohepatitis, CPA Collagen Proportionate Area
